# Circular RNA expression profile in blood according to ischemic stroke etiology

**DOI:** 10.1186/s13578-020-00394-3

**Published:** 2020-03-10

**Authors:** Aiora Ostolaza, Idoia Blanco-Luquin, Amaya Urdánoz-Casado, Idoya Rubio, Alberto Labarga, Beatriz Zandio, Miren Roldán, Judith Martínez-Cascales, Sergio Mayor, María Herrera, Nuria Aymerich, Jaime Gallego, Roberto Muñoz, Maite Mendioroz

**Affiliations:** 1 Department of Neurology, Complejo Hospitalario de Navarra-IdiSNA (Navarra Institute for Health Research), 31008 Pamplona, Navarra Spain; 2 Neuroepigenetics Laboratory-Navarrabiomed-IdiSNA, Complejo Hospitalario de Navarra, Universidad Pública de Navarra (UPNA), IdiSNA (Navarra Institute for Health Research), C/Irunlarrea, 3, 31008 Pamplona, Navarra Spain; 3 Stroke Unit, Department of Neurology, Complejo Hospitalario de Navarra- IdiSNA (Navarra Institute for Health Research), 31008 Pamplona, Navarra Spain; 4 Bioinformatics Unit, Navarrabiomed, Public University of Navarre (UPNA), IdiSNA (Navarra Institute for Health Research), C/Irunlarrea, 3, 31008 Pamplona, Navarra Spain

**Keywords:** Stroke, Etiology, circRNA, miRNA, Biomarker, *UBA52*

## Abstract

**Background:**

The discovery of novel biomarkers of stroke etiology would be most helpful in management of acute ischemic stroke patients. Recently, circular RNAs (circRNAs) have been proposed as candidate biomarkers of neurological conditions due to its high stability. circRNAs function as sponges, sequestering miRNAs and are involved in most relevant biological functions. Our aim was to identify differentially expressed circRNAs in acute ischemic stroke patients according to stroke etiology.

**Methods:**

A comprehensive expression profile of blood circRNAs was conducted by Arraystar Human circRNA arrays (13,617 probes) on a discovery cohort of 30 stroke patients with different stroke etiologies by TOAST classification. Real-time quantitative PCR (RT-qPCR) was used to validate array results in a cohort of 50 stroke patients. Functional in silico analysis was performed to identify potential interactions with microRNAs (miRNAs) and pathways underlying deregulated circRNAs.

**Results:**

A set of 60 circRNAs were found to be upregulated in atherotrombotic versus cardioembolic strokes (fold-change > = 1.5 and p-value ≤ 0.05). Differential expression of hsa_circRNA_102488, originated from *UBA52* gene, was replicated in the validation cohort. RNA-binding proteins (RBPs) sites of hsa_circRNA_102488 clustered around AGO2 and FUS proteins. Further functional analysis revealed interactions between deregulated circRNAs and a set of miRNAs involved in stroke-related pathways, such as fatty acid biogenesis or lysine degradation.

**Conclusion:**

Different stroke subtypes show specific profiles of circRNAs expression. circRNAs may serve as a new source of biomarkers of stroke etiology in acute ischemic stroke patients.

## Introduction

Stroke is a common vascular disease that causes death and disability and is therefore a major challenge to current healthcare systems [1]. Nowadays, stroke diagnosis is still based on clinical criteria and imaging data, so clinicians are able to identify the etiology in about 75–80% of stroke cases by following the TOAST classification system [2]. In the remaining 20–25% of cases, the exact cause is unknown. It has been estimated that in about 25–30% of these cause-undetermined events, the underlying source of stroke could be paroxysmal atrial fibrillation (PAF) [1]. However, demonstrating this or other causes behind undetermined stroke events still remains a challenge in the clinical setting. Therefore, there is an increasing need for biomarkers capable of identifying stroke etiology in clinical practice. In recent years, numerous studies have investigated a large amount of new blood biomarkers in relation with stroke etiology [3–7]. However, low sensitivity and specificity of the target biomarkers difficult their translation into clinical practice. Thus, the discovery of new molecules to aid the diagnosis of stroke etiology would be most helpful in this scenario.

By far, proteins represent the most widely studied class of molecules in the identification of biomarkers for cerebrovascular diseases. However, RNA molecules were also suggested as candidate stroke biomarkers about a decade ago [8, 9]. In particular, brain and blood profiling in rat models revealed differential expression of certain microRNAs (miRNAs) after transient focal ischemia [8] and blood miRNAs were proposed as diagnostic and prognostic biomarkers in acute stroke patients [9].

Since then, a wide range of RNA species have been discovered, including circular RNAs (circRNAs). circRNAs are endogenous single-stranded RNA molecules which form a circle through a covalent binding [10]. These molecules are evolutionary conserved and very abundant in the human transcriptome [11]. circRNAs display a wide range of regulatory functions in RNA biology and gene expression. For instance, some circRNAs function as sponges that sequester miRNAs or RNA-binding proteins, regulating the expression of target genes [12]. In addition, given that circRNAs do not have free ends, they are resistant to endonuclease activity and therefore more stable than other linear RNA species such as messenger RNA (mRNA) or even microRNAs (miRNAs) [13]. This features make circRNAs promising biomarkers in different medical conditions. Indeed, circRNAs have recently been proposed as potential clinical biomarkers for neurological disorders [14]. However, comprehensive circRNAs expression has not been examined in stroke patients so far.

In this study, we have comprehensively profiled circRNAs in the peripheral blood of ischemic stroke patients during the acute stage. To this end, we used Arraystar Human circRNA Array (8 × 15 K, Arraystar), which surveys up to 13,617 probes to identify human circRNAs, in 30 acute stroke patients with different etiologies by TOAST classification [2]. As a result, we show that distinct circRNAs are differentially expressed in atherotrombotic *versus* cardioembolic stroke patients, arising as promising candidate biomarkers of stroke etiology.

## Methods

### Study population

Patients with acute ischemic stroke admitted to the emergency department of the Hospital of Navarra within the first 4.5 h after symptoms onset were included in the study. Among a cohort of 700 consecutive patients recruited from January 2015 to December 2016, 30 patients were included in the discovery cohort (Table 1) and 50 patients were included in the validation cohort (Additional file 2: Table S1). All participants completely fulfilled the etiology testing protocol (see methods below). Informed consent was obtained from all participants and the study was approved by the local Ethics Committee.

**Table 1:** Demographic and clinical characteristics of patients

	Atherothrombotic (n = 8)	Cardioembolic (n = 14)	Undetermined (n = 8)	p-value
Age—years, median (IQR)	70 (55–80)	75 (70.5–77)	66.5 (49–77)	0.366
Male, n (%)	7 (87.5)	7 (50)	5 (62.5)	0.214
High blood pressure, n (%)	6 (75)	12 (85.7)	3 (37.5)	0.056
Diabetes mellitus, n (%)	2 (25)	2 (14.3)	2 (25)	0.765
Dyslipidemia, n (%)	3 (37.5)	9 (64.3)	5 (62.5)	0.441
Smoker, n (%)	4 (50)	2 (20)	3 (42.9)	0.38
Cardiopathy, n (%)	3 (37.5)	6 (42.9)	0 (0)	0.093
Atrial fibrillation, n (%)	0 (0)	15 (100)	0 (0)	< 0.001***
Peripheral arteropathy, n (%)	2 (25)	0 (0)	0 (0)	0.053
Basal mRankin, median (IQR)	0.5 (0–1)	0 (0–1.25)	0 (0–0.75)	0.565
Basal NIHSS, median (RIQ)	8.5 (5–18)	20 (17–22)	19 (18–20)	0.049*
Significant ipsilateral carotid stenosis (%)	8 (100)	0 (0)	1 (14.3)	< 0.001***
Hemorrhagic transformation, n (%)	5 (62.5)	4 (28.6)	2 (25)	0.206
Discharge mRankin, median (IQR)	4.5 (2–6)	4 (2–5)	3 (0.5–5)	0.434

*IRQ* interquartile range

### Clinical, vascular and brain imaging protocol

A detailed history of vascular risk factors including hypertension, atrial fibrillation, diabetes mellitus, dyslipidemia, tobacco, cardiovascular disease and peripheral atherosclerosis was recorded for each patient. In order to determine stroke etiology, a set of tests was performed including electrocardiogram (EKG), chest radiography, complete blood count, blood biochemistry analysis, carotid ultrasonography, transcranial doppler (TCD) examination, non-contrast cranial tomography (CT) at baseline, echocardiogram and 24 h holter monitoring. Patients were classified into etiological subgroups according to Trial of Org 10172 in Acute Stroke Treatment (TOAST) criteria [2].

### Microarray expression of circRNAs

Venous blood samples were drawn from acute stroke patients within 24 h after admission. Total RNA was isolated from blood samples using miRNeasy Mini kit (QIAGEN, Redwood City, CA, USA) which enables purification of total RNA, including RNA from approximately 18 nucleotides upwards, following manufacturer’s instructions. Genomic DNA was removed with a recombinant DNase (TURBO DNA-free™ Kit, Ambion, Inc., Austin, TX, USA). RNA integrity was assessed by electrophoresis on a denaturing agarose gel. Concentration of RNA was determined by OD260 using a NanoDrop ND-1000 spectrophotometer. Array star Human circRNA Array V2 analysis (Arraystar, Inc., Rockville, MD, USA) of the 30 selected stroke samples was performed as follows. Sample labeling and array hybridization were completed according to the manufacturer’s protocol (Arraystar Inc.). Briefly, 2000 ng of total RNAs were digested with RNase R (Epicentre, Inc.) to remove linear RNAs and enrich circular RNAs. Then, the enriched circular RNAs were amplified and transcribed into fluorescent cRNA utilizing a random priming method (Arraystar Super RNA Labeling Kit; Arraystar). The labeled cRNAs were purified by RNeasy Mini Kit (Qiagen) and hybridized onto the Arraystar Human circRNA Array V2 (8 × 15 K, Arraystar). After having washed the slides, the arrays were scanned by the Agilent Scanner G2505C (Agilent Technologies, Inc., Santa Clara, CA, USA).

### circRNAs microarray data

Scanned images were imported into Agilent Feature Extraction software (version 11.0.1.1) for raw data extraction. Quantile normalization of raw data and subsequent data processing were performed using the R software (R Project for Statistical Computing, Vienna, Austria) limma package. Normalized intensity values are shown in Additional file 1: Fig. S1. After quantile normalization of the raw data, low intensity filtering was performed, and those circRNAs in which at least 8 out of 30 samples had flags in “P” or “M” (“All Targets Value”) were retained for further analyses. Three distinct groups by stroke etiology (cardioembolic, atherotrombotic and undetermined) were analyzed by pairs, so three different comparisons were performed. When comparing two groups of profile differences (such as cardioembolic *versus* atherotrombotic), the fold change (FC) (i.e. the ratio of the group averages) between the groups for each circRNA was computed. Differentially expressed circRNAs between two groups were identified through FC filtering and statistical significance of the difference between two groups was estimated by Student’s t-test. CircRNAs having FC ≥ 1.5 and p-values ≤ 0.01 were selected as significantly differentially expressed. Differentially expressed circRNAs with statistical significance between two groups were shown by Scatter plots and Volcano plots.

### Validation of array results by qPCR

Blood RNA isolated by miRNeasy Mini kit (QIAGEN, Redwood City, CA, USA) from the validation cohort (n = 50) was used to validate array results. Complementary DNA (cDNA) was reverse transcribed from 500 ng total RNA per sample with SuperScript^®^ III First-Strand Synthesis Reverse Transcriptase (Invitrogen, Carlsbad, CA, USA) and primed with random primers. Real time-qPCR (RT-qPCR) reactions were performed in triplicate for each sample using Power SYBR^®^ Green PCR Master Mix (Invitrogen, Carlsbad, CA, USA) in a QuantStudio 12 K Flex Real-Time PCR System (Applied Biosystems, Foster City, CA, USA). Divergent primer pairs were designed to overlap the backspliced junction by using Primer3 website (http://primer3.ut.ee/). Furthermore, *GAPDH* housekeeping gene and convergent primer pairs to amplify the host mRNA were designed by Real Time PCR tool (IDT, Coralville, IA, USA). Primer sequences are listed in Additional file 2: Table S2. At the designing stage, verification of primers specificity was carried out by PCR tool at the UCSC Genome Browser [15]. After amplification, we also checked that RT-qPCR reaction had generated a single-peak in the melting curve and a single amplicon of the correct size by performing agarose gel electrophoresis. The thermal cycling conditions consisted of an initial denaturation step at 95 °C for 10 min followed by 40 cycles of 15 s at 95 °C and 1 min at 60 °C. Expression levels of each corresponding linear transcript, *GAPDH* or convergent amplicon of the host gene, was used to normalize circRNA levels [16]. Relative expression level of circRNA in a particular sample was calculated by the delta delta-CT method, as previously described [17]. Non-template reactions were included as negative controls in each run. Finally, qPCR amplicons were subjected to Sanger sequencing [18] and checked for the presence of the predicted backspliced junctions in order to test their circularity.

### Functional in silico analysis

Since certain circRNAs may function as sponges sequestering miRNAs and, therefore, may be involved in the regulation of gene expression, it was interesting to explore the potential interactions between differentially expressed circRNAs in stroke and miRNAs. The circRNA/microRNA interaction was predicted with Arraystar’s home-made miRNA target prediction software based on TargetScan & miRanda [19], and the differentially expressed circRNAs were annotated in detail with the circRNA/miRNA interaction information. Then, overrepresented miRNAs (those linked to at least four differentially expressed circRNAs) were analyzed by DIANA-mirPath v.3 software [20] to predict the underlying pathways.

CircInteractome tool provided a list of miRNAs potentially targeted by the circRNA of interest and mapped binding sites for RNA-binding proteins (RBPs) on it [21]. Next, DIANA-mirPath v.3 software [20] analyzed the pathways in which the outcome miRNAs were involved based on TarBase v7.0, microT-CDS v5.0 and TargetScan. Kyoto Encyclopedia of Genes and Genomes (KEGG) and Gene Ontology (GO) analyses were used to predict cell signaling pathways and functions related with the set of outcome miRNAs.

Moreover, in order to assess the interactions betweem the RBPs predicted to bind a particular differentially expressed circRNA, protein list was uploaded to the Search Tool for the Retrieval of Interacting Genes (STRING) tool (Version 11.0) [22] and filtered for interactions of high confidence (score > 0.7) and PANTHER (Protein ANalysis THrough Evolutionary Relationships) Classification System (Version 14.1) [23]. Only those terms with a FDR-corrected p-value < 0.05 were reported.

### Statistical Analysis

Statistical analysis was performed with SPSS 21.0 (IBM, Inc., USA). First, adjustment to normal distribution was tested for all continuous variables as per one-sample Kolgomorov-Smirnov test and the normal quantil-quantil (QQ) plots. Data represents the mean ± standard deviation (SD) or the median (interquartile range). In the discovery cohort, univariate analysis of demographic and clinical characteristics was performed by ANOVA or Kruskall-Wallis test along with Chi square test. In the validation cohort, univariate analysis of demographic and clinical characteristics was performed by Student t-test or U Mann–Whitney test along with Chi square test. circRNA expression differences between two groups was estimated by Mann–Whitney U test. For all the comparisons, significance level was set at p-value < 0.05. SPSS 21.0 (IBM, Inc., USA) was used to draw graphs.

## Results

### Differential expression of circRNAs in etiologic stroke subtypes

To begin to ask whether circRNAs were differentially expressed in ischemic stroke depending on subtype etiology, we performed Arraystar Human circRNA Array V2 analysis, which included 13,617 distinct probes for human circRNAs, on a target group of 30 patients suffering from acute ischemic stroke with different etiologies according to TOAST classification [2], i.e. 14 cardioembolic, 8 atherotrombotic and 8 undetermined stroke. Demographic and clinical characteristics of patients included in the discovery cohort are shown in Table 1. Normalized intensity values showed similar distributions of the intensities (expression values) for all samples (Additional file 1: Fig. S1).

**Fig. 1:**
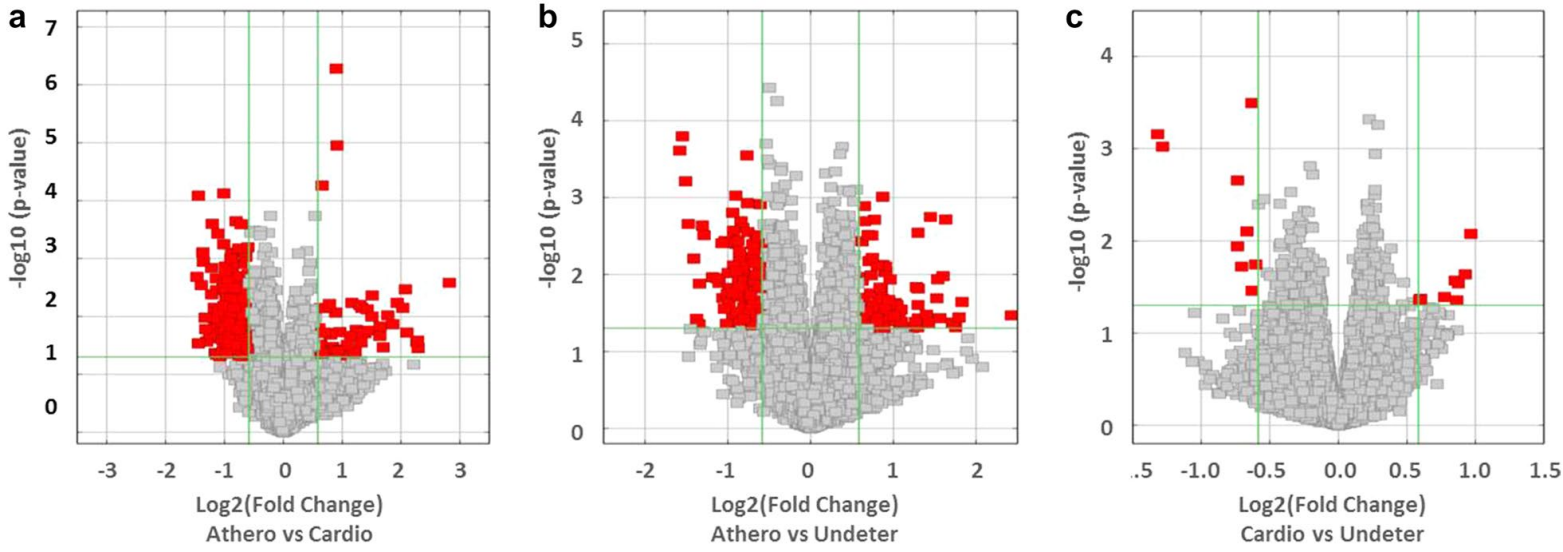
Volcano-plots of differential expression of circRNAs for stroke etiology comparisons. Graphs visualize the relationship between fold-change (magnitude of change) in the X axes and statistical significance (which takes both magnitude of change and variability into consideration) in the Y axes. A high number of circRNAs are shown as differentially expressed between atherotrombotic versus cardioembolic strokes (**a**), and atherotrombotic versus undetermined strokes (**b**) whereas cardioembolic versus undetermined strokes show less differences suggesting both groups are similar in terms of circRNA expression. The vertical green lines correspond to the threshold of 1.5-fold up and down, respectively, and the horizontal green line represents a p-value = 0.05. Red points in the plot represent the differentially expressed circRNAs with statistical significance which crossed the fold-change threshold

The main aim of this study was to identify differences in circRNA expression between atherotrombotic and cardioembolic stroke patients. We found 219 differentially expressed circRNAs (FC ≥ 1.5 and p-value ≤ 0.05) in atherotrombotic *versus* cardioembolic stroke patients (Additional file 2: Tables S3 and S4). circRNA expression variation between the two compared groups is represented as volcano plots and scatter plots (Fig. 1a, Additional file 1: Fig. S2A). Despite we observed a higher number of downregulated (159) than upregulated (60) circRNAs in atherotrombotic compared to cardioembolic samples, the best findings in terms of statistical significance and magnitude of change were found among the upregulated circRNAs. Indeed, 11.7% (7) of upregulated circRNAs showed more than fourfold change in expression (Additional file 2: Table S3).

When comparing atherotrombotic versus undetermined strokes, 226 circRNAs were found to be differentially expressed (FC ≥ 1.5 and p-value ≤ 0.05), being 87 circRNAs upregulated and 139 circRNAs downregulated in atherotrombotic compared to undetermined strokes (Additional file 2: Tables S5 and S6, Fig. 1b and Additional file 1: Fig. S2B). In this set, differences were not as strong as in the previous comparison of atherotrombotic *versus* cardioembolic strokes, and only one circRNA showed expression higher than fourfold change. Finally, a very few changes were found when comparing cardioembolic and undetermined strokes, as only 8 circRNAs were found to be upregulated and 9 circRNAs were downregulated in this comparison (Additional file 2: Table S7, Fig. 1c and Additional file 1: Fig. S2C).

**Fig. 2:**
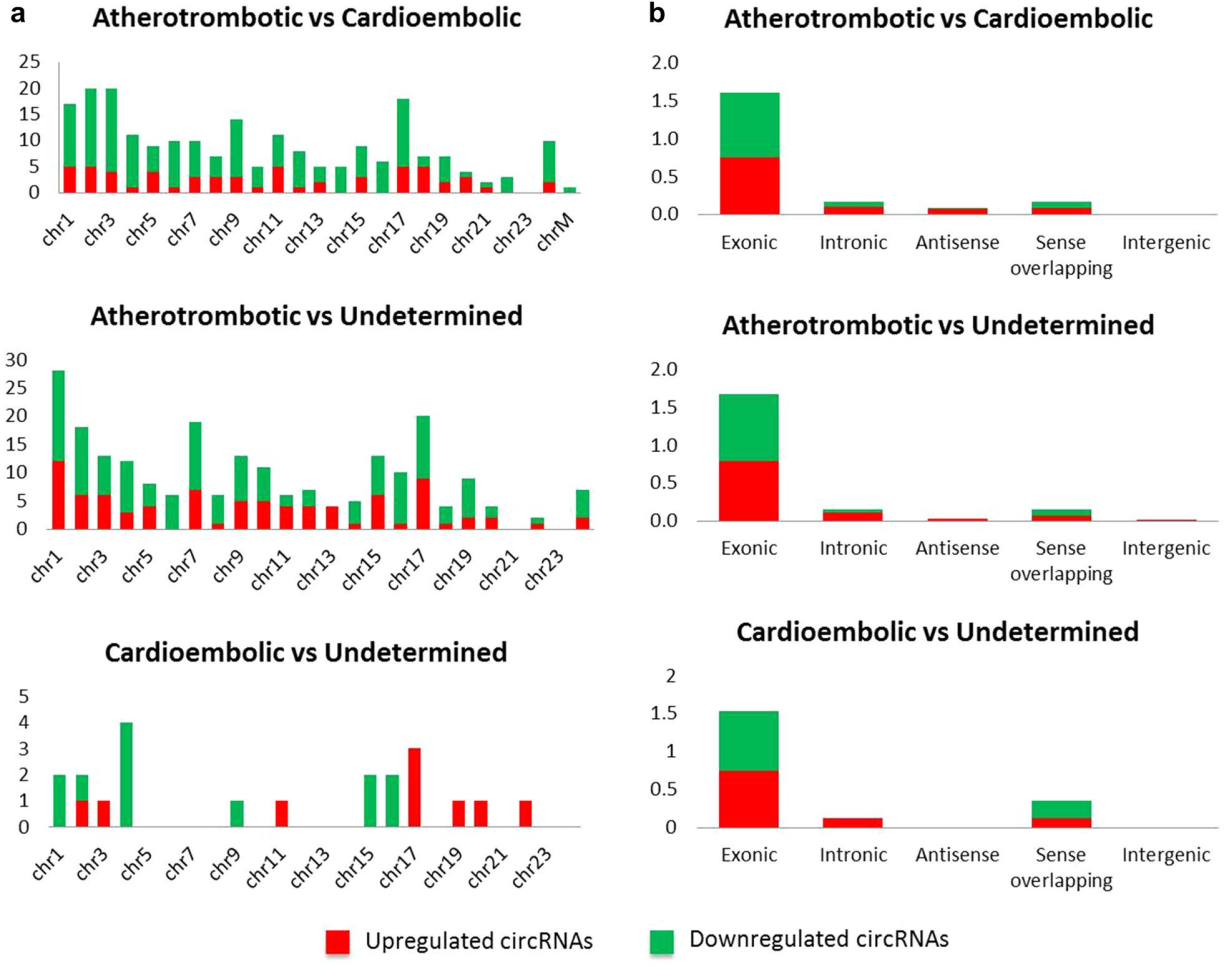
Distribution by chromosomes (**a**) and chromosome’s region (**b**). Differentially expressed circRNA distribution in chromosomes and chromosome´s region (exonic, intronic, antisense, sense overlapping and intergenic) for each comparison

As shown in Fig. 2A, differentially expressed circRNAs seem to cluster in chromosomes 1, 2, 3 and 17 for the comparison atherotrombotic *versus* cardioembolic stroke (> 15 hits); 1, 2, 7 and 17 for atherotrombotic *versus* undetermined (> 15 hits) and 4 and 17 for cardioembolic *versus* undetermined (> 2.5 hits). With these results in mind, circRNAs from chromosome 17, and even 1 and 2, appear to be important for stroke etiology. In terms of distribution by chromosome regions, the number of differentially expressed circRNAs that are transcribed from protein-coding exons is the most abundant and comparable across downregulated and upregulated circRNAs (Fig. 2b). There were only a few intronic, antisense, sense overlapping and intergenic type circRNAs.

### Validation of differentially expressed circRNAs by RT-qPCR

**Fig. 3:**
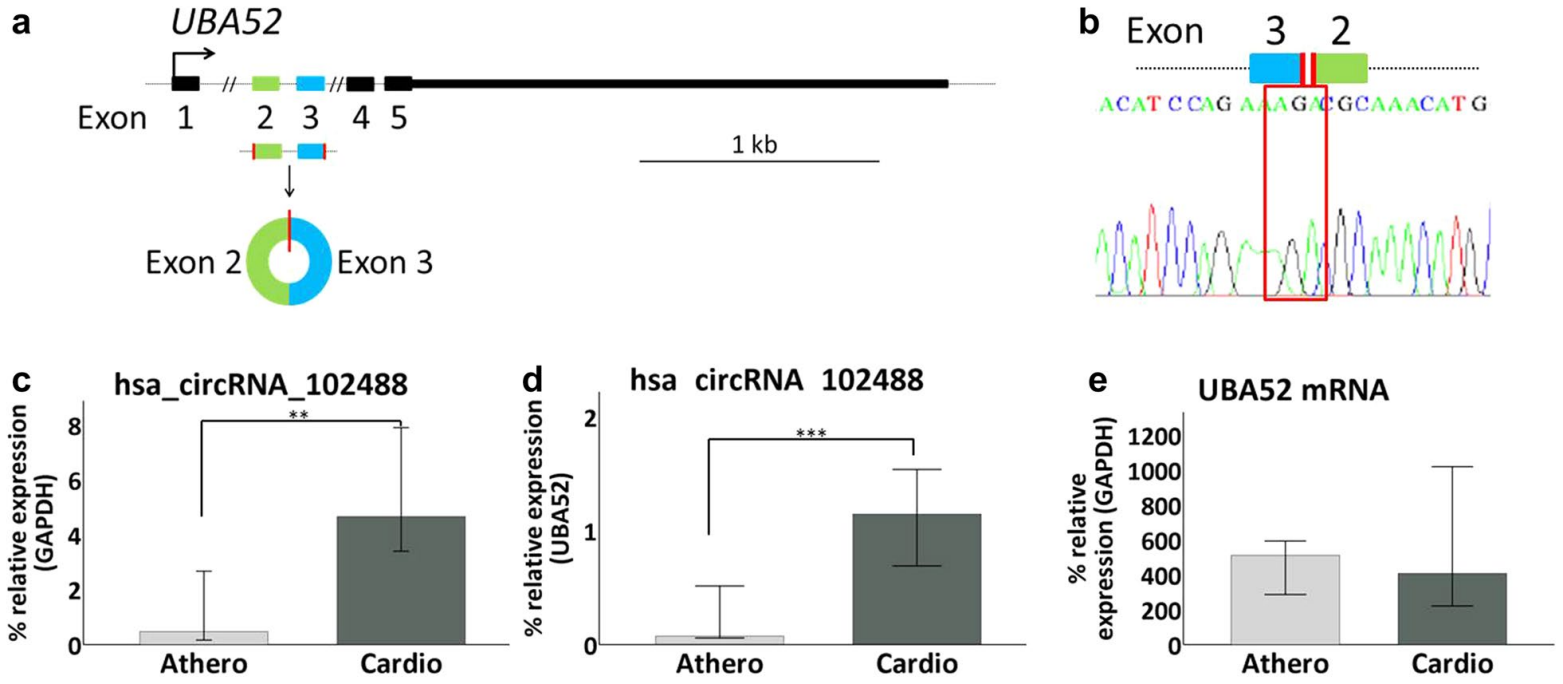
RT-qPCR validation of hsa_circRNA_102488. **a** Schematic representation of the *UBA52* gene, origin of hsa_circRNA_102488. The graph shows the map of hsa_circRNA_102488 which is originated from exons 2 and 3 of its host mRNA (NCBI RefSeq track shown in the UCSC Genome Browser). **b** hsa_circRNA_102488 backsplicing. The Sanger-Sequencing electropherogram specifies the sequence of the backspliced junction that results in the circRNA formation. **c**–**e** hsa_circRNA_102488 and *UBA52* mRNA expression levels between atherotrombotic vs cardioembolic samples. Percentage of hsa_circRNA_102488 expression relative to *GAPDH* mRNA (**c**) or *UBA52* mRNA (**d**) is decreased in atherotrombotic with respect to cardioembolic stroke patients. However, we found similar expression levels of *UBA52* mRNA in atherotrombotic and cardioembolic samples groups (**e**). Error bars: 95% CI. **p-value < 0.01; ***p-value < 0.001

To validate the differences in circRNA expression, we focused on the atherotrombotic versus cardioembolic comparison since that was the main goal of our study. An expanded cohort which included samples previously analyzed in the microarray (n = 50; 25 atherotrombotic and 25 cardioembolic samples) was used to this end. Next, 3 upregulated and 2 downregulated circRNAs in atherotrombotic *versus* cardioembolic stroke patients were selected based on highest fold change and/or lowest p-value prioritization (Table 2). Then, we performed RT-qPCR to amplify and quantify the chosen circRNAs, their host mRNAs and *GAPDH* mRNA levels. Compared to the linear transcript, only hsa_circRNA_102488 showed a statistically significant change in expression between etiology subtypes. hsa_circRNA_102488 is originated from the ubiquitin a-52 residue ribosomal protein fusion product 1 (*UBA52*) gene which is located at chromosome 19 (Fig. 3a). Sanger sequencing confirmed the presence of the backsplicing junction between *UBA52*´ exons 3 and 2 (Fig. 3b). Mann–Whitney U test revealed that expression levels of hsa_circRNA_102488 (alias hsa_circ_0005568) were lower for atherotrombotic compared to cardioembolic samples when normalizing to *GAPDH* mRNA (p-value < 0.01) (Fig. 3c), as well as to the corresponding *UBA52* mRNA (p-value < 0.001) (Fig. 3d). Interestingly, we did not found significant differences in *UBA52* mRNA expression levels between atherotrombotic and cardioembolic stroke samples (Fig. 3e).

**Table 2:** Deregulated circRNA chosen for qPCR validation

Regulation	CircRNA	CircRNA type	FC (abs)	P-value	Chromosome position			Gene symbol
Up	hsa_circRNA_001359	intronic	7.0479384	0.002611197	chr1	169663839	169664181	SELL
	hsa_circRNA_103559	exonic	4.8769307	0.035206182	chr3	196118683	196120490	UBXN7
	hsa_circRNA_104220	exonic	4.8572966	0.026033314	chr6	150092297	150094305	PCMT1
Down	hsa_circRNA_102488	exonic	2.7229035	8.17917E−05	chr19	18684102	18684558	UBA52
	hsa_circRNA_104748	exonic	2.3292681	0.001448543	chr9	20907148	20929595	FOCAD

*CircRNA* deregulated circRNA with greater intensity values in atherotrombotic stroke patients compared with cardioembolic; circRNA_type: the circRNAs are classified into 5 types: “exonic”, “intronic”, “antisense”, “sense overlapping” and “intergenic”; *FC* absolute ratio (no log scale) of normalized intensities between two conditions; *p-value* p-value calculated from unpaired t-test; Annotations, include chromosome position and Gene Symbol

### Functional in silico analysis of differentially expressed circRNAs

**Fig. 4:**
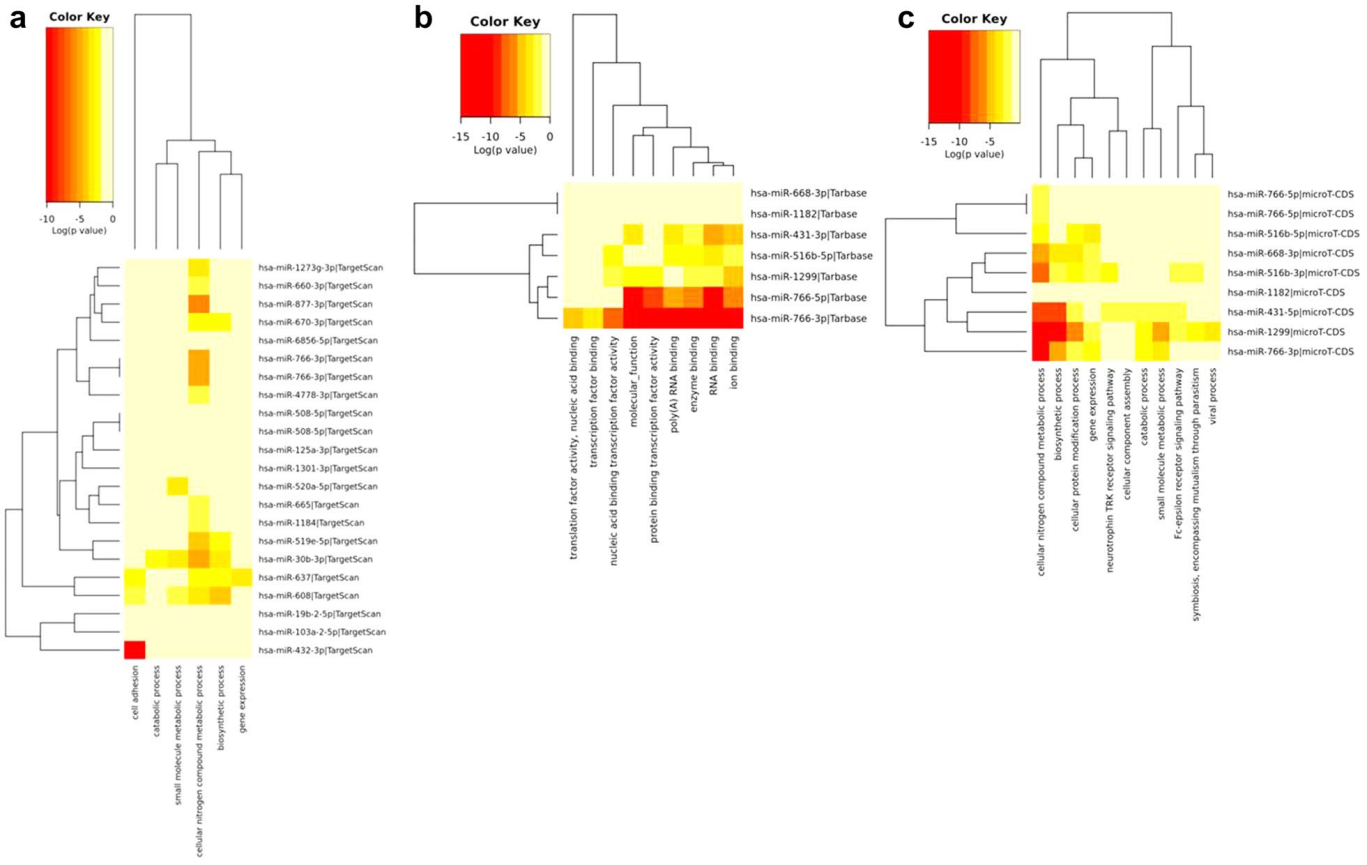
GO analysis by mirPath v.3. The image shows the heatmaps corresponding to the analysis performed with the overrepresented miRNAs in the three comparisons (atherotrombotic-cardioembolic-undetermined) by TargetScan (**a**) and the analysis involving the target miRNAs for hsa_circRNA_102488 in accordance with molecular function (**b**, by Tarbase) or biological process (**c**, by microT-CDS)

In order to explore potential interactions between differentially expressed circRNAs in stroke subtypes and target miRNAs, a bioinformatics analysis was performed (see “Methods”). First, interactions between circRNAs and their target miRNAs were predicted by Arraystar’s home-made software. Then, we selected the overrepresented miRNAs in the three comparisons (those miRNAs linked to at least four differentially expressed circRNAs), and characterized their related pathways by DIANA-mirPath (p-value threshold ≤ 0.05). Overrepresented miRNAs were associated with fatty acid biosynthesis and metabolism, lysine degradation, arrhythmogenic right ventricular cardiomyopathy (ARVC), adrenergic signaling in cardiomyocytes or hypertrophic cardiomyopathy (HCM) as shown by KEGG analysis (p-value threshold < 0.05) (Additional file 1: Fig. S3A). Moreover, these miRNAs were linked to cellular nitrogen compound metabolic process, homophilic cell adhesion via plasma membrane adhesion molecules, cell adhesion, blood coagulation or neurotrophin tyrosine-kinase (TRK) receptor signaling pathway in GO analysis (p-value threshold < 0.05) (Fig. 4a).

Regarding the validated circRNA, hsa_circRNA_102488, CircInteractome tool [21] showed one 7mer-m8 or 7mer-1a type target site shared by the following miRNAs: hsa-miR-1182, hsa-miR-1299, hsa-miR-431, hsa-miR-516b, hsa-miR-668 and hsa-miR-766. DIANA-mirPath analysis revealed that this set of miRNAs converged on the pathways of fatty acid biosynthesis and metabolism, extracellular matrix (ECM)-receptor interaction, lysine degradation or arrhythmogenic right ventricular cardiomyopathy (ARVC) as shown by KEGG analysis (p-value threshold < 0.001 (Additional file 1: Fig. S3B, C). They seemed to have a common function as RNA-, ion- and enzyme-binding molecules, a protein and nucleic acid binding transcription factor activity and to be involved in cellular nitrogen compound metabolic, biosynthetic, cellular protein modification and gene expression processes (GO analysis, p-value threshold < 0.05) (Fig. 4b, c).

In addition, up to 10 RBPs sites matching to hsa_circRNA_102488 were identified by CircInteractome: 3 for SFRS1, 2 for AGO2, HuR, IGF2BP2 and PTB and 1 for CAPRIN1, DGCR8, FMRP, IGF2BP1 and LIN28B. STRING clustered these RBPs around AGO2 (protein–protein interaction, PPI, enrichment p-value < 1.0e−16) (Additional file 1: Fig. S4A–C) and PANTHER related them to two categories of molecular functions: RNA binding and catalytic activity. Moreover, other RBPs sites that matched to the hsa_circRNA_102488 flanking regions were identify for *EIF4A3* (9 Tags), *HNRNPC*, *HuR*, *U2AF65* and *FUS* genes. After functional analysis, we observed that these RBPs clustered around FUS (PPI enrichment p-value < 2.11e−10) and related to catalytic activity, IGF2BP1 and IGF2BP3 to binding molecular function and ZC3H7B separately but also to catalytic activity function (STRING and PANTHER tools) (Additional file 1: Fig. S4D–F).

**Fig. 5:**
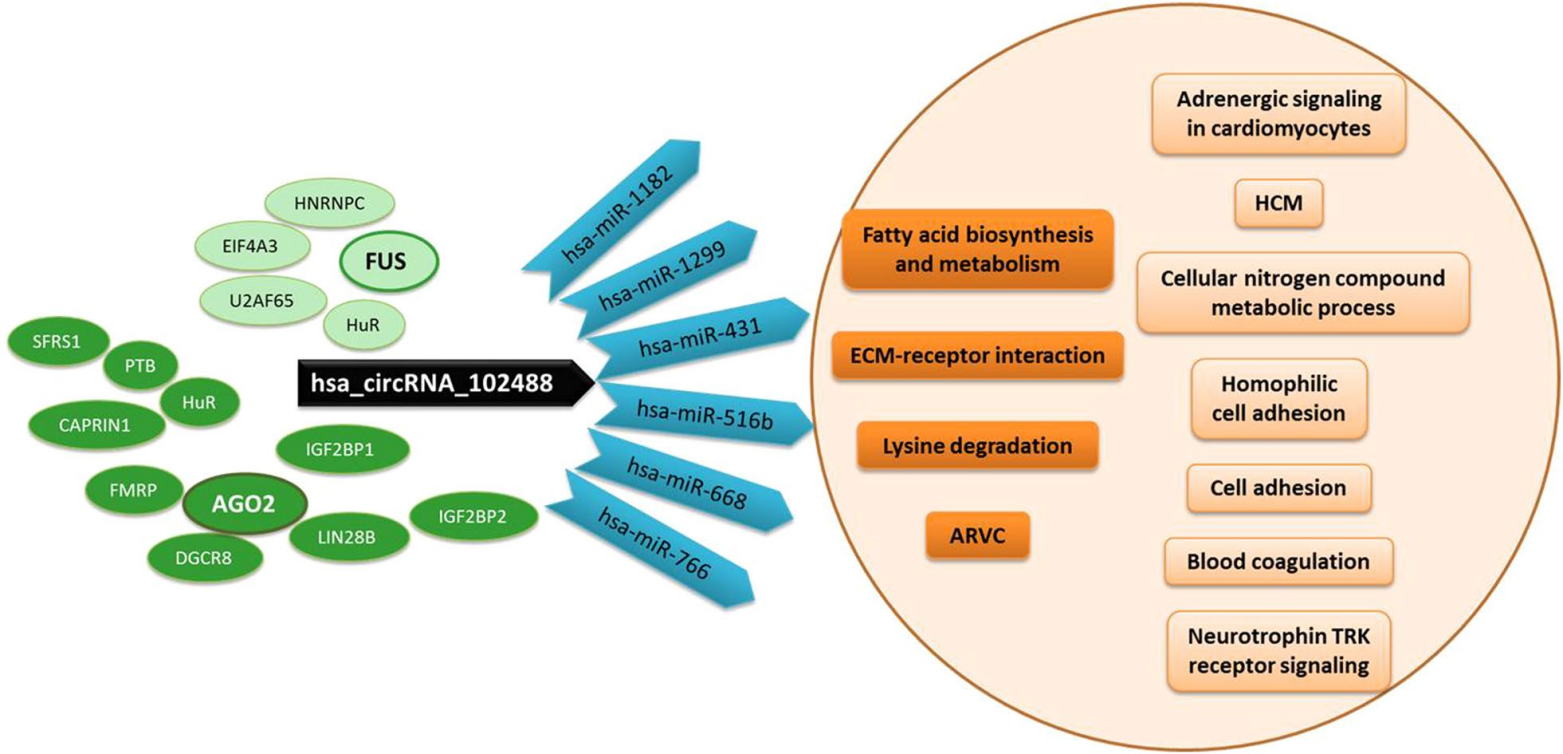
hsa_circRNA_102488 impact pathways. The cartoon shows miRNAs (in blue) sharing a target site with hsa_circRNA_102488 (in black) and the pathways in which they converge (in dark orange), within those associated with the overrepresented miRNAs in the three comparisons (in light orange). RBPs sites matching to hsa_circRNA_102488 clustered around AGO2 (in dark green) and RBPs sites that matched to its flanking regions clustered around FUS (in light green). ARVC: Arrhythmogenic Right Ventricular Cardiomyopathy; HCM: Hypertrophic Cardiomyopathy

A diagram illustrating impact pathways mentioned above is shown in Fig. 5.

## Discussion

In this study, we identified circRNAs that were differentially expressed in human blood according to stroke etiology. The strongest differences were found for the comparison between atherotrombotic and cardioembolic stroke. Differentially expressed circRNAs were predicted to predominantly interact with a set of miRNAs involved in stroke-related pathways, including fatty acid biosynthesis, lysine degradation, arrhythmogenic right ventricular cardiomyopathy (ARVC) or hypertrophic cardiomyopathy (HCM), among others.

circRNAs are a new class of non-coding RNA molecules with circular morphology due to the formation of a covalent junction between the 3′ and 5′ ends. These molecules are generated from a parental (also known as host) pre-messenger RNA through a particular alternative splicing process known as backsplicing [24, 25]. The covalent bond confers resistance to ribonucleases preventing degradation and making circRNAs very stable molecules. Furthermore, circRNAs are expressed in most mammalian tissues, particularly in the brain and have been also detected in the human blood [26–28]. All these characteristics make circRNAs potential biomarkers of human diseases and, especially, of neurological diseases.

Indeed, previous reports suggesting the potential role of circRNAs as biomarkers or therapeutic targets in neurological conditions, such as multiple sclerosis or epilepsy, have been already published [29–31]. In the stroke field, a number of changes in circRNAs expression levels have been recently described. In 2017, it was observed for the first time that circRNAs are altered in the mouse brain after transient focal ischemia [32, 33]. Despite these initial observations, as far as we know, a comprehensive profiling of circRNAs in human stroke or its relationship to stroke etiology has not previously been reported.

In our study, a set of 219 differentially expressed circRNAs between atherotrombotic and cardioembolic stroke was found in the blood of acute stroke patients. The most robust statistical differences were observed among the 60 circRNAs up-regulated in atherotrombotic compared to cardioembolic samples. Most of them tended to cluster in chromosome 17 and were of exonic origin, which is not surprising since the majority of known circRNA molecules are exonic and therefore, there is an overrepresentation of exonic circRNAs in the Arraystar microarray.

Even though a large number of circRNAs were shown to be differentially expressed in atherotrombotic *versus* undetermined stroke samples, there were hardly any differences between cardioembolic and undetermined strokes. This result suggests that the undetermined stroke group may harbor a great proportion of non-recognized cardioembolic strokes, which is in line with current knowledge in the clinical setting. In fact, it has been described that some patients with stroke of undetermined etiology may have atrial structural or functional changes thus increasing the risk of cardioembolism [34].

In recent years, functions of circRNAs are beginning to be understood. It has been observed that circRNAs can act at different levels of gene regulation, including transcription, mRNA splicing or translation. However, the best known function so far is the regulation of gene expression by acting as miRNA sponges: circRNAs have binding sites for specific miRNAs and act by “sequestering” them to prevent miRNAs from exerting translational repression. Thus far, a few stroke-related circRNAs acting as miRNA sponges have been observed. circRNA DLGAP4 functions as a sponge for miRNA-143 and therefore, inhibits miRNA-143 activity. Interestingly, overexpression of circRNA DLGAP4 ameliorates neurological deficit and infarct volume after transient focal ischemia in a stroke mouse model. circRNA DLGAP4 seems to significantly attenuate blood–brain barrier damage by regulating tight junction protein expression and endothelial-mesenchymal transition in endothelial cells [35]. Other example of deregulated circRNAs in stroke is circRNA Hectd1, which was found to be upregulated in the brain of mice after transient middle cerebral artery occlusion (tMCAO). circRNA HECTD1 participates in the inhibition of astrocyte activation via macroautophagy/autophagy by acting as a sponge for MIR-142 [36]. Interestingly, circRNA HECTD1 was increased in peripheral blood mononuclear cells from acute ischemic stroke patients compared to controls and higher levels of circRNA HECTD1 were related with stroke recurrence [37]. The circRNA TLK1, which acts as an endogenous miR-335-3p sponge, has been proved to be detrimental in ischemic brain injury. It was increased in the mouse brain after tMCAO and knocking down circRNA TLK1 improve neurological deficits and infarct volume in this stroke mouse model. Remarkably, circRNA TLK1 was observed to be also elevated in human patients after ischemic stroke [38].

In the present study, differentially expressed circRNAs were predicted to interact with numerous miRNAs. Functional study showed that those miRNAs were involved in pathways associated with stroke etiology or pathogenesis, such as fatty acids biogenesis, lysine degradation or development of cardiopathy, including ARVC and HCM. Elevated levels of free fatty acids have been associated with cardioembolic etiology of stroke irrespective of the presence of atrial fibrillation [39, 40] and predict stroke recurrence in patients with atrial fibrillation [41]. Although the underlying mechanisms remain unclear, fatty acids are major components of epicardial fat and it is known that thickness of epicardial fat is related with the presence of atrial fibrillation [42]. According to that, serum fatty acid binding proteins are proposed as biomarkers for atrial fibrillation [43]. However, fatty acids seem independent predictors of stroke events so they may be directly related to thrombogenesis [39, 40].

Interestingly, lysine degradation pathway showed a strong association with overrepresented miRNAs in our study. Lysine is an essential aminoacid with pleiotropic functions in humans. In addition to proteinogenesis, lysine is involved in the crosslinking of collagen peptides, the uptake of calcium and iron and is the precursor of carnitine, which in turn is essential in the metabolism of fatty acids [44]. Lysine degradation differs in brain from that of in extracerebral tissues and it is known to be regulated by thyroid hormones [45]. For now, its role in stroke is not yet well understood. A recent study among hypertensive subjects revealed altered metabolic pathways, including lysine degradation, related to incident ischemic stroke [46]. Moreover, a metabolomic study showed that levels of serum lysine catabolites were low in patients at high-risk of suffering ischemic stroke [47].

Despite ARVC is not among the classical embolic sources of stroke events, ARVC pathways have been recently associated with ischemic stroke in a comprehensive long non-coding RNA transcriptomic study [48] and this is consistent with our results. HCM is a common hereditary cardiomyopathy frequently associated with sudden death. It has been described that atrial fibrillation with risk for progressive heart failure and embolic stroke occurs in 20% of patients with HCM [49].

In this study, we were able to validate hsa_circRNA_102488 in an expanded cohort of atherotrombotic versus cardioembolic strokes. This circRNA is formed from the pre-mRNA of *UBA52* gene and has a 456 bp genomic length. hsa_circRNA_102488 was found to be highly expressed in brain tissues [27]. No previous records of the association between *UBA52* gene and stroke have been reported to our knowledge.

Functional analysis revealed that hsa_circRNA_102488 harbors a binding site for 6 different miRNAs. Intriguingly, this set of 6 miRNAs is enriched in pathways similar to those found for the overrepresented miRNAs, namely fatty acids biogenesis, lysine degradation and ARVC. This result suggests that hsa_circRNA_102488 may be representative of the altered circRNA network and its related pathways underlying stroke etiology and makes hsa_circRNA_102488 an interesting candidate worth to be explored in futures studies.

Among known functions, circRNAs interact with RNA binding proteins (RBPs) to influence gene transcription and translation [50]. In hsa_circRNA_102488, the analysis revealed RBPs binding sites that cluster around two RBPs: argonaute 2 (Ago2) and RNA-binding protein FUS. Argonaute 2 is a critical component of the RNA-induced silencing complex (RISC) and, as a consequence, a master regulator of miRNAs-dependent gene silencing pathway. Interestingly, it has been observed that Ago2 accumulates and undergoes hydroxylation following hypoxia, thus Ago2 emerges as a major regulator of the pathological responses to hypoxia [51]. In fact, stroke substantially modifies Ago2-associated miRNA profiles in the brain in a stroke rat model [52]. On the other hand, FUS participates in DNA repair mechanisms [53] and is frequently mutated in amyotrophic lateral sclerosis [54]. However, no previous works on the role of FUS in stroke has been previously reported.

The determination of circRNA as biomarkers in the etiology of stroke would be a great advance in the diagnostic process of cerebral vascular diseases. It should be noted that circRNAs are especially interesting as biomarkers because they represent a gene regulatory mechanism for several genes at once and, despite being RNA molecules, they are extremely stable. A large part of the study carried out on these patients is aimed to find the cause of the stroke, and in up to 25% of cases a clear etiology cannot be established. The risk factors that predispose to presenting an atherothrombotic stroke are widely known and current guidelines make general recommendations to prevent them. However, patients who remain without a specific etiological diagnosis would not benefit from these guidelines. Moreover, knowing the cause of stroke in a rapid and non-invasive way would help to improve secondary prevention and avoid unnecessary studies.

As a discovery study, where typically a large number of molecules are measured in a reduced number of subjects, an obvious limitation of this study is the limited sample size. Therefore, we should be cautious with our conclusions. For the sake of external validity, these findings should be replicated in blood samples of an independent cohort of ischemic stroke patients with different etiologies.

## Conclusions

The present study showed a set of differentially expressed circRNAs in peripheral blood from different stroke etiologic subtypes. These circRNAs revealed interesting pathways underlying stroke etiology, such as fatty acid biogenesis or lysine degradation. Due to their molecular features, circRNAs may be useful as candidate biomarkers for stroke etiology. Large and independent cohort studies will be needed to further investigate the role of circRNAs as stroke biomarkers.

## Supplementary information

**Additional file 1.** Additional figures.

**Additional file 2.** Additional tables.

## Data Availability

The datasets used and/or analysed during the current study are available from the corresponding author on reasonable request.
