# Supplementary material for: Circular RNA expression profile in blood according to ischemic stroke etiology
Source: Cell Biosci. 2020 Mar 10;10:34. doi: 10.1186/s13578-020-00394-3 (PMC7063791; doi:10.1186/s13578-020-00394-3)
Supplement: Supplementary file 1 — Additional file 1. Additional figures. [file 13578_2020_394_MOESM1_ESM.pdf]

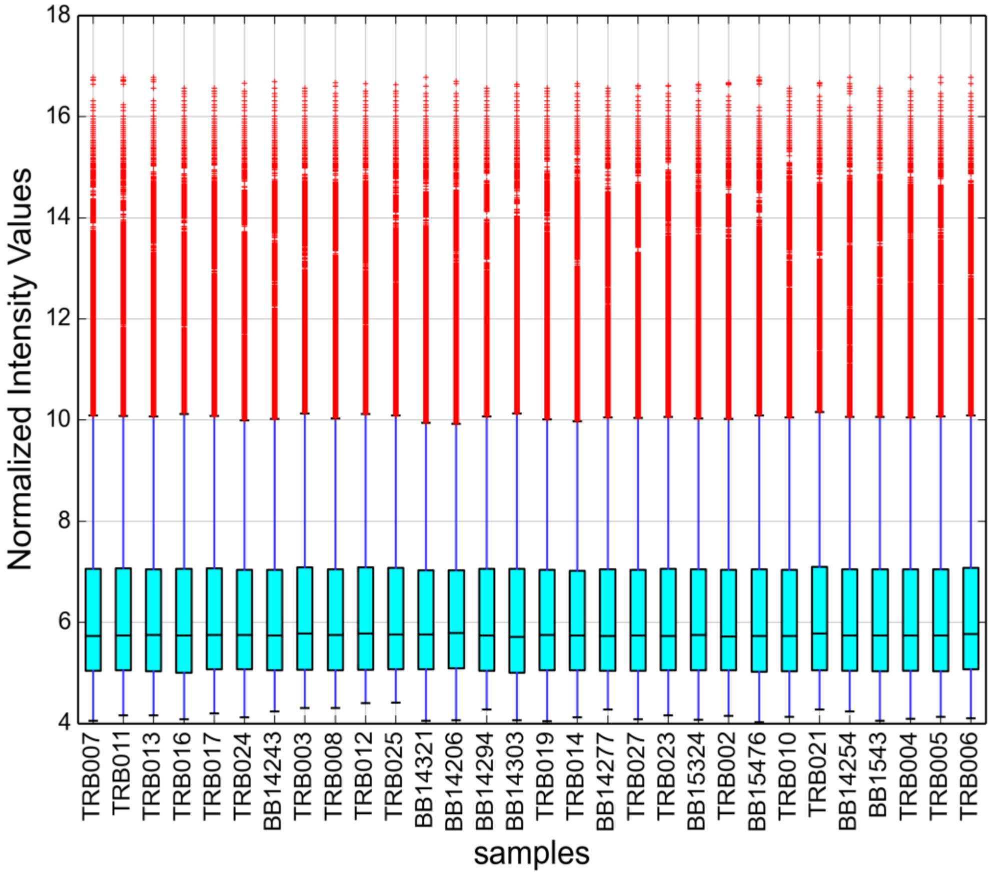

Additional figure S1. Box Plot – CircRNAs. The Box Plot graph depicts the distribution of the dataset from the Arraystar Human circRNA Array V2 in acute stroke patients after normalization. The graph shows similar distributions of the intensities (expression values) from all samples.

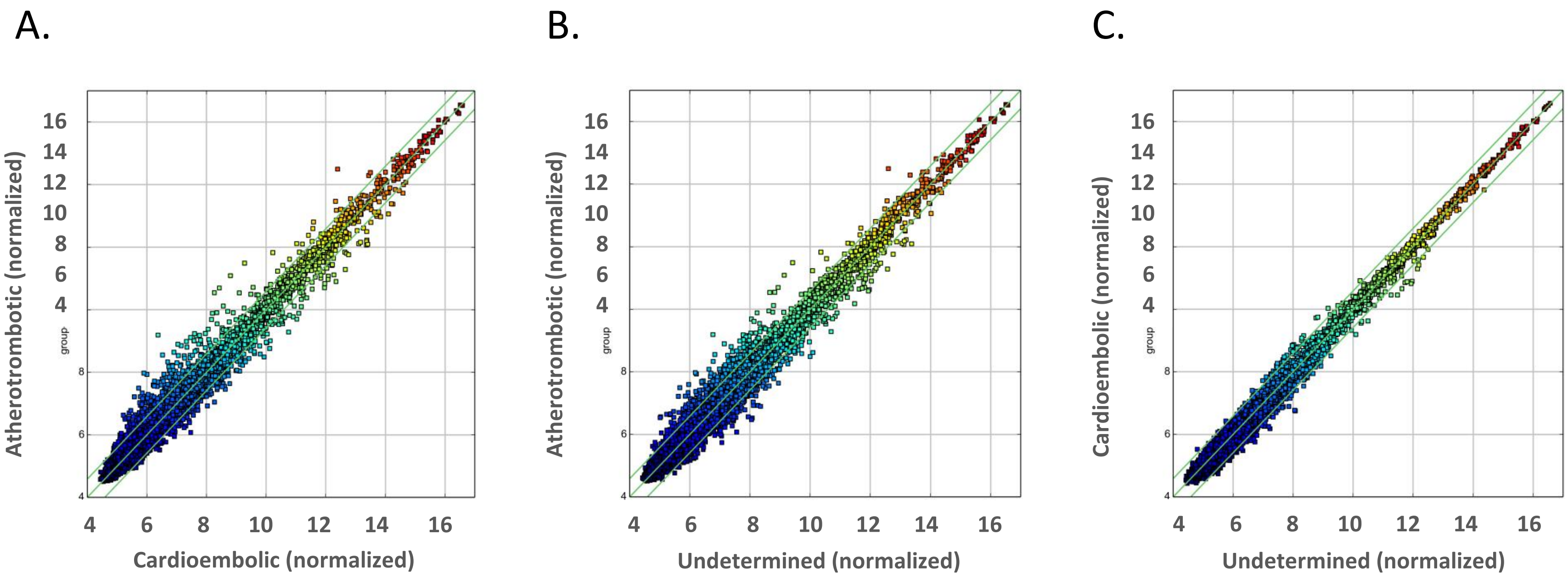

Additional figure S2: Scatter-Plots of circRNA signal values for stroke etiology comparisons. The values of X and Y axes in the Scatter-Plots show the averaged normalized signal values of groups of samples (log2 scaled). Light green lines represent the fold-change threshold. The circRNAs above the top green line and below the bottom green line indicated more than 1.5 fold change of circRNAs expression between atherotrombotic and cardioembolic (A), atherotrombotic and undetermined (B) and cardioembolic and undetermined (C) groups of patients.

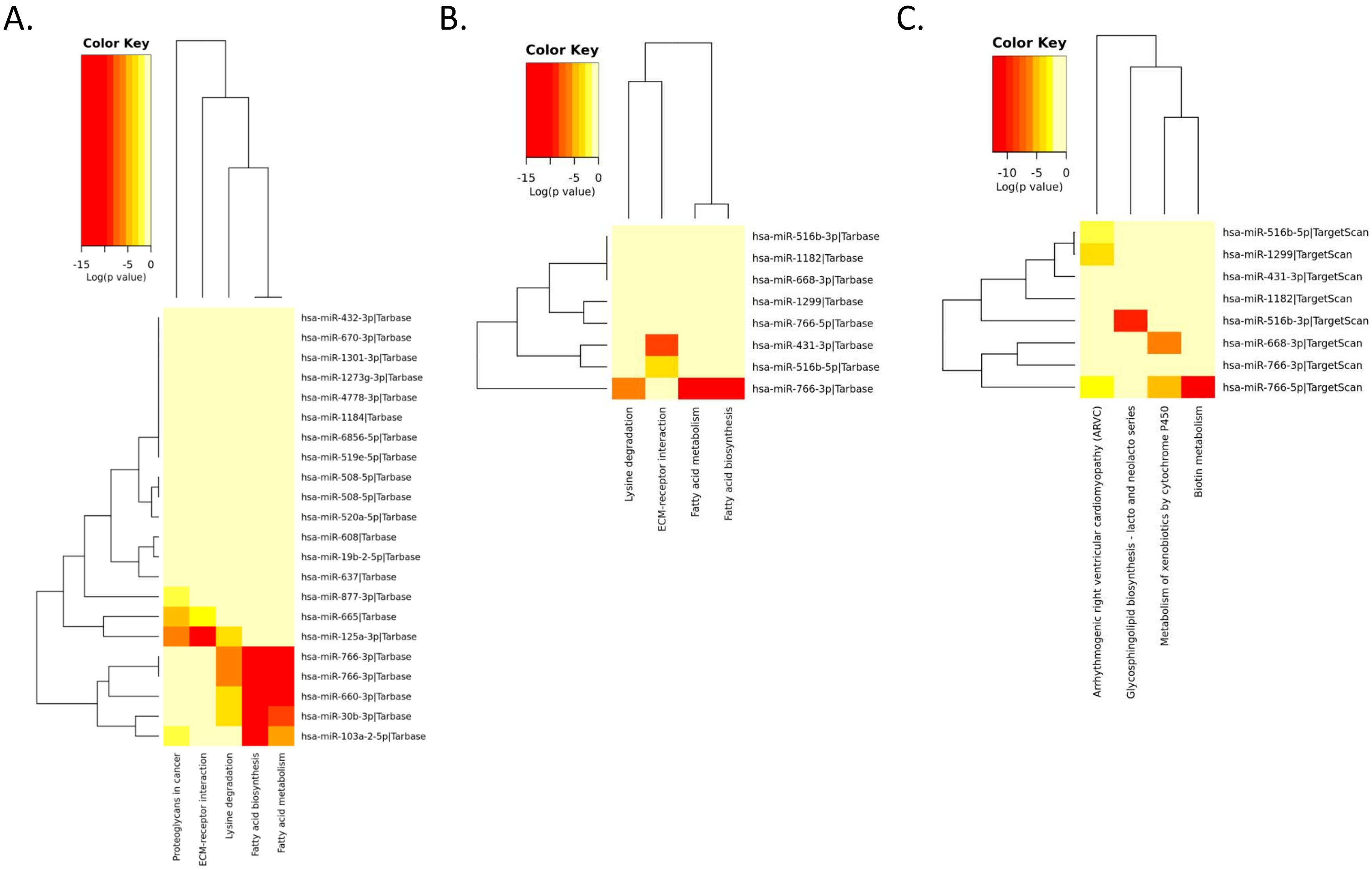
A.
Color Key
-15
-5
0
Log(p value)
hsa-miR-432-3p|Tarbase
hsa-miR-670-3p|Tarbase
hsa-miR-1301-3p|Tarbase
hsa-miR-1273g-3p|Tarbase
hsa-miR-4778-3p|Tarbase
hsa-miR-1184|Tarbase
hsa-miR-6856-5p|Tarbase
hsa-miR-519e-5p|Tarbase
hsa-miR-508-5p|Tarbase
hsa-miR-508-5p|Tarbase
hsa-miR-520a-5p|Tarbase
hsa-miR-608|Tarbase
hsa-miR-19b-2-5p|Tarbase
hsa-miR-637|Tarbase
hsa-miR-877-3p|Tarbase
hsa-miR-665|Tarbase
hsa-miR-125a-3p|Tarbase
hsa-miR-766-3p|Tarbase
hsa-miR-766-3p|Tarbase
hsa-miR-660-3p|Tarbase
hsa-miR-30b-3p|Tarbase
hsa-miR-103a-2-5p|Tarbase
Proteoglycans in cancer
ECM-receptor interaction
Lysine degradation
Fatty acid biosynthesis
Fatty acid metabolism
B.
Color Key
-15
-5
0
Log(p value)
hsa-miR-516b-3p|Tarbase
hsa-miR-1182|Tarbase
hsa-miR-668-3p|Tarbase
hsa-miR-1299|Tarbase
hsa-miR-766-5p|Tarbase
hsa-miR-431-3p|Tarbase
hsa-miR-516b-5p|Tarbase
hsa-miR-766-3p|Tarbase
Lysine degradation
ECM-receptor interaction
Fatty acid metabolism
Fatty acid biosynthesis
C.
Color Key
-10
-5
0
Log(p value)
hsa-miR-516b-5p|TargetScan
hsa-miR-1299|TargetScan
hsa-miR-431-3p|TargetScan
hsa-miR-1182|TargetScan
hsa-miR-516b-3p|TargetScan
hsa-miR-668-3p|TargetScan
hsa-miR-766-3p|TargetScan
hsa-miR-766-5p|TargetScan
Arrhythmogenic right ventricular cardiomyopathy (ARVC)
Glycosphingolipid biosynthesis - lacto and neolacto series
Metabolism of xenobiotics by cytochrome P450
Biotin metabolism

Additional figure S3. KEGG Analysis. The heatmaps show the KEGG Pathway involving the overrepresented miRNAs in the three comparisons (atherotrombotic-cardioembolic-undetermined) by Tarbase (A) and the ones resulted with target miRNAs for hsa_circRNA_102488 by Tarbase (B) and TargetScan (C).

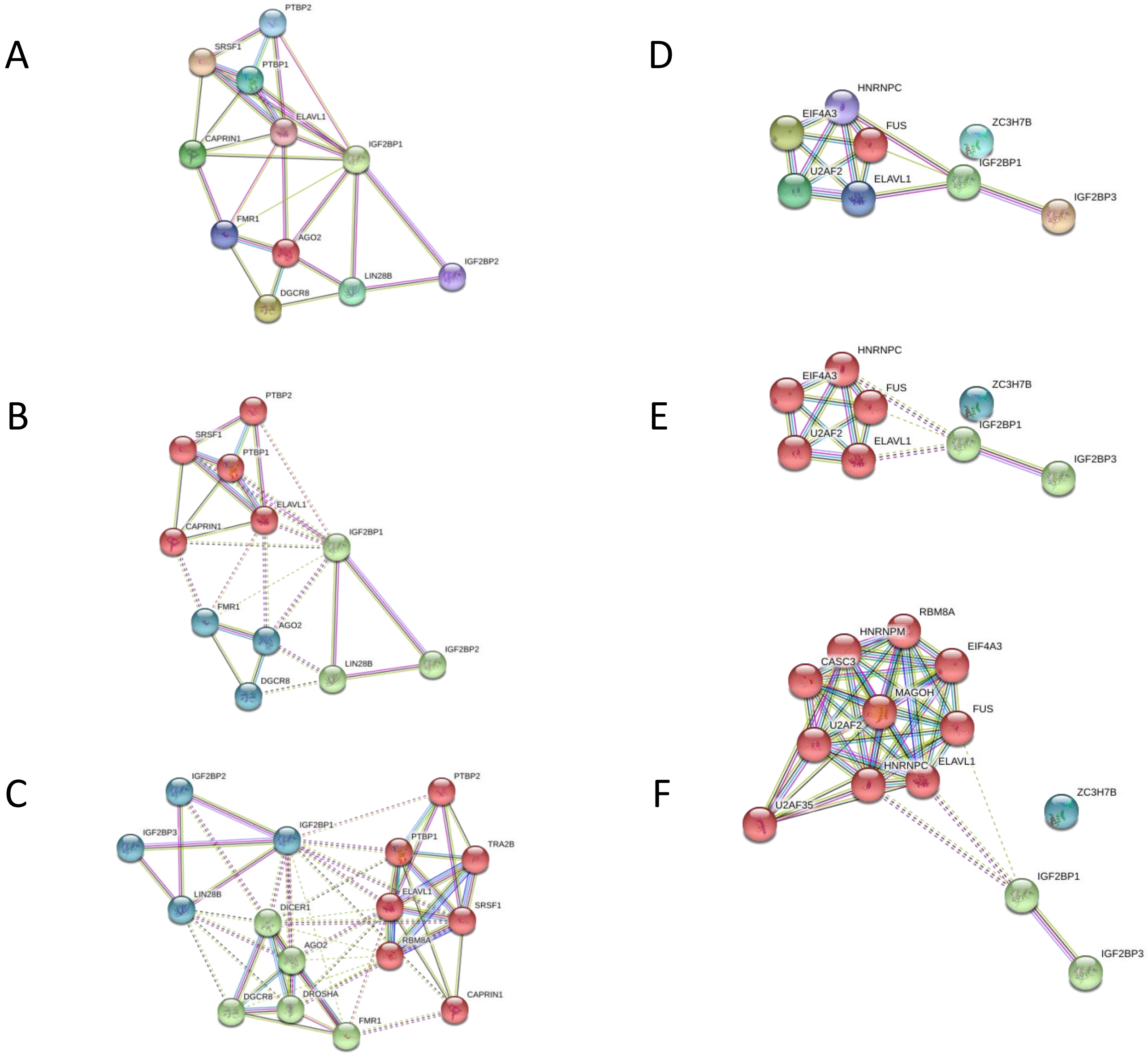

Additional figure S4. Functional protein association networks constructed by STRING. The analysis shows the clustered RBPs sites matching hsa_circRNA_102488 (A), the network clustering (B) and more nodes added to the network and the clustered RBPs flanking regions (D), its network clustering (E) and more added nodes (F).
