# Supplementary material for: Circular RNA expression profile in blood according to ischemic stroke etiology
Source: Cell Biosci. 2020 Mar 10;10:34. doi: 10.1186/s13578-020-00394-3 (PMC7063791; doi:10.1186/s13578-020-00394-3)
Supplement: Supplementary file 2 — Additional file 2. Additional tables. [file 13578_2020_394_MOESM2_ESM.pdf]

Additional table S1. Demographic and clinical characteristics of patients included in the validation cohort.

	Atherothrombotic (n=25)	Cardioembolic (n=25)	p-value
Age – years, mean (SD)	73.7 (9.7)	73.8 (9.9)	0.904
Male, n (%)	17 (68)	17 (68)	1.000
High blood pressure, n (%)	16 (64)	17 (68)	0.765
Diabetes mellitus, n (%)	9 (36)	5 (20)	0.208
Dyslipemia, n (%)	15 (60)	14 (56)	0.774
Smoker, n (%)	7 (28)	4 (16)	0.383
Cardiopathy, n (%)	4 (16)	9 (36)	0.107
Previous atrial fibrillation, n (%)	0 (0)	12 (48)	7.10E-05
Peripheral arteropathy, n (%)	8 (32)	1 (4)	0.010
Basal mRankin, median (IQR)	0 (0-3)	1 (0-3)	0.281
Basal NIHSS, median (RIQ)	4 (0-20)	4 (1-23)	0.585
Significant ipsilateral carotid stenosis (%)	16 (64)	0 (0)	3.00E-06
Hemorrhagic transformation, n (%)	4 (16)	5 (20)	0.368
Discharge mRankin, median (IQR)	2 (0-5)	2.28 (0-6)	0.945

Additional table S2. RT-qPCR primers. The table shows the primer pairs used in the study. Divergent primers amplify the circular form and convergent primers amplify the linear transcript. Amplified transcripts are identified by RefSeq Accession or GeneBank accesion number.

	circRNA (Alias)		Divergent primers	Tm	Amplicon (bp)	Convergent primers	Tm	Amplicon (bp)
UPREGULATED	hsa_circRNA_001359 (hsa_circ_0000157)	Forward:	TTTCATTTCTCTTTCCCTTAGGTG	60	126	CCTCTTCATTCCAGTGGCAG	62.3	131
		Reverse:	GAGACAGAACAAGCAGTCATCTC	58.2		CAAGGGCGATTTAATATGGGTC	61.2	
	hsa_circRNA_103559 (hsa_circ_0005051)	Forward:	CAGTCTGAAAATCCCGGAAA	60.04	149	GTGCCCCAATTCCTCAAAAG	61.5	150
		Reverse:	TGGAGGAGCTATCGATAAGAAA	58.11		CATTTCTTAATTCTTGTTCTTGCCG	61.5	
	hsa_circRNA_104220 (hsa_circ_0006936)	Forward:	AAGCAACAATCAGTGCTCCA	59.45	114	AGCTAATCCACAATCTCCGC	62.0	100
		Reverse:	CCATGTATGGGTTACATTTTGC	59.12		GGGTTACATTTTGCATAGTGGG	61.6	
DOWNREGULATED	hsa_circRNA_102488 (hsa_circ_0005568)	Forward:	CTGACCAGCAGCGTCTGATA	60.16	121	AGACCCTCACTGGCAAAAC	62.1	124
		Reverse:	TTTTGCCAGTGAGGGTCTTC	60.23		CCGGCAAATATCAGACGCTG	63.1	
	hsa_circRNA_104748 (hsa_circ_0008508)	Forward:	AAGAGAAACTCAGGTTCATATCCA	58.35	148	GCTTGCATACATGAATCGAGC	61.8	147
		Reverse:	AAAATGGCATGATAAGCTCGAT	59.96		GTCAGCATGTCTCTAACCCAG	62.0	
Housekeeping	GAPDH	Forward:	n.a.	n.a.	n.a.	ACATCGCTCAGACACCATG	61.8	143
		Reverse:	n.a.	n.a.		TGTAGTTGAGGTCAATGAAGGG	61.9	

qPCR: quantitative PCR; bp: base pair; Tm: Melting Temperature; n.a.: not applicable.

Additional table S3. Upregulated expression of top 25 circRNAs in atherotrombotic versus cardioembolic stroke patients. 1.5 Fold up regulated circRNAs with a 0.05 p-value cut-off in the condition pair Atherotrombotic versus Cardioembolic.

circRNA	p-value	FC (abs)	Alias (circBase)	chromosome position			circRNA_type	GeneSymbol
hsa_circRNA_101978	0.015966166	1.5197703	hsa_circ_0005869	chr17	8347577	8363478	exonic	NDEL1
hsa_circRNA_100837	0.014830014	3.1180593	hsa_circ_0022537	chr11	62594626	62595103	exonic	STX5
hsa_circRNA_043650	0.038889828	1.8017644	hsa_circ_0043650	chr17	39845126	39847898	exonic	EIF1
hsa_circRNA_047274	0.016933446	2.3595931	hsa_circ_0047274	chr18	21645975	21663045	exonic	TTC39C
hsa_circRNA_000110	0.004329165	2.8448915	hsa_circ_0000110	chr1	114372213	114377061	exonic	PTPN22
hsa_circRNA_102034	0.027628257	4.8107589	hsa_circ_0005397	chr17	30500849	30503232	exonic	RHOT1
hsa_circRNA_060102	0.043195894	1.6417483	hsa_circ_0060102	chr20	34143977	34145405	exonic	ERGIC3
hsa_circRNA_008069	0.047642389	1.5207656	hsa_circ_0008069	chr3	133341934	133343019	exonic	TOPBP1
hsa_circRNA_407205	0.009558362	3.4126921		chr9	99481790	99483412	sense overlapping	RP11-535M15.1
hsa_circRNA_102894	0.034124787	3.2382664	hsa_circ_0001092	chr2	202010100	202014558	exonic	CFLAR
hsa_circRNA_000855	0.007110478	1.549565	hsa_circ_0000632	chr15	74927307	74929309	sense overlapping	EDC3
hsa_circRNA_103621	0.019447239	1.5798282	hsa_circ_0001402	chr4	38091552	38104778	exonic	TBC1D1
hsa_circRNA_002011	0.046882763	2.1264235	hsa_circ_0000606	chr15	60734614	60737990	sense overlapping	ICE2
hsa_circRNA_071668	1.11383E-05	1.8788448	hsa_circ_0071668	chr5	843714	843839	exonic	ZDHHC11
hsa_circRNA_407340	5.46112E-05	1.5758504		chrX	138844119	138857112	exonic	ATP11C
hsa_circRNA_103559	0.035206182	4.8769307	hsa_circ_0005051	chr3	196118683	196120490	exonic	UBXN7
hsa_circRNA_080099	0.018754085	2.5315418	hsa_circ_0080099	chr7	45011271	45016256	exonic	MYO1G
hsa_circRNA_008868	0.022829233	1.5482839	hsa_circ_0008868	chr13	53196123	53201713	exonic	HNRNPA1L2
hsa_circRNA_002414	5.2483E-07	1.8622187	hsa_circ_0002414	chr1	202385914	202391866	exonic	PPP1R12B
hsa_circRNA_103456	0.005812954	2.2392393	hsa_circ_0067127	chr3	125843206	125856803	exonic	ALDH1L1
hsa_circRNA_102562	0.039854144	1.7662311	hsa_circ_0051258	chr19	42482087	42482950	exonic	ATP1A3
hsa_circRNA_086154	0.03362912	1.7828057	hsa_circ_0086154	chr8	145806229	145806661	exonic	ARHGAP39

hsa_circRNA_402509	0.007080814	2.5139679		chr2	242033691	242036842	exonic	MTERF4
hsa_circRNA_019664	0.008520785	1.8487716	hsa_circ_0019664	chr10	103868785	103871293	exonic	LDB1
hsa_circRNA_100760	0.019499945	2.6109279	hsa_circ_0021150	chr11	9228219	9229179	exonic	DENND5A

circRNA: deregulated circRNA with greater intensity values in atherotrombotic stroke patients compared with cardioembolic; p-value: p-value calculated from unpaired t-test; FC (abs): absolute ratio (no log scale) of normalized intensities between two conditions; Alias: circRNA ID in circBase (http://circbase.mdc-berlin.de); Annotations, include chrom, txStart, txEnd, circRNA_type and GeneSymbol; circRNA_type: The circRNAs are classified into 5 types: "exonic", "intronic", "antisense", "sense overlapping" and "intergenic". "Exonic" represents circRNA arising from the exons of the linear transcript; "Intronic" represents the circRNA arising from an intron of the linear transcript; "antisense" represents circRNA whose gene locus overlap with the linear RNA, but transcribed from the opposite strand; "sense overlapping" represents circRNA transcribed from same gene locus as the linear transcript, but not classified into "exonic" and "intronic"; "intergenic" represents circRNA located outside known gene locus.

Additional table S4. Downregulated expression of top 25 circRNAs in atherotrombotic versus cardioembolic stroke patients. 1.5 Fold up regulated circRNAs with a 0.05 p-value cut-off in the condition pair Atherotrombotic versus Cardioembolic.

circRNA	p-value	FC (abs)	Alias (circBase)	chromosome position			circRNA_type	GeneSymbol
hsa_circRNA_104195	0.042479371	1.502024	hsa_circ_0002198	chr6	136472297	136476896	exonic	PDE7B
hsa_circRNA_100018	0.000776361	2.582232	hsa_circ_0009361	chr1	1749275	1770677	exonic	GNB1
hsa_circRNA_091722	0.015633339	1.5918225	hsa_circ_0091722	chrX	151934651	151936377	exonic	MAGEA3
hsa_circRNA_403834	0.01632827	1.5339036		chr7	72863871	72865324	exonic	BAZ1B
hsa_circRNA_092416	0.00166963	1.8223009	hsa_circ_0000593	chr15	41988272	42005694	exonic	MGA
hsa_circRNA_044097	0.036072196	1.8233658	hsa_circ_0044097	chr17	42929776	42932372	exonic	EFTUD2
hsa_circRNA_001795	0.005134167	1.6339025	hsa_circ_0001795	chr8	42294506	42323435	exonic	SLC20A2
hsa_circRNA_004646	0.008710966	1.6339243	hsa_circ_0004646	chr1	162546566	162567648	exonic	UAP1
hsa_circRNA_075625	0.004313447	1.7122911	hsa_circ_0075625	chr6	10935290	10956475	exonic	SYCP2L
hsa_circRNA_403472	0.022748181	1.5983493		chr5	142675024	142680328	exonic	NR3C1
hsa_circRNA_100604	0.015639701	1.5852606	hsa_circ_0009172	chr10	70218860	70229920	exonic	DNA2
hsa_circRNA_028152	0.00350283	2.1942846	hsa_circ_0028152	chr12	110397651	110399490	exonic	GIT2
hsa_circRNA_083776	0.000790999	1.8255029	hsa_circ_0083776	chr8	27514298	27530537	exonic	SCARA3
hsa_circRNA_104126	0.008454177	1.8010017	hsa_circ_0076798	chr6	53365044	53365148	exonic	GCLC
hsa_circRNA_405133	0.020788003	1.5684033		chr13	36939636	36943737	sense overlapping	SPG20-AS1
hsa_circRNA_004745	0.033840209	1.6207475	hsa_circ_0004745	chr17	33932723	33935406	exonic	AP2B1
hsa_circRNA_103639	0.012656692	1.7289701	hsa_circ_0007646	chr4	52729602	52758017	exonic	DCUN1D4
hsa_circRNA_405450	0.012985157	1.6532237		chr16	19576172	19592975	exonic	C16orf62
hsa_circRNA_000480	0.015950555	2.2300805	hsa_circ_0000480	chr13	46559797	46563115	antisense	ZC3H13
hsa_circRNA_003898	0.014631252	1.6356215	hsa_circ_0003898	chr2	231624673	231658046	exonic	CAB39
hsa_circRNA_091000	0.018760587	1.7152249	hsa_circ_0091000	chrX	70514068	70521018	exonic	NONO
hsa_circRNA_101732	0.029026864	2.0281138	hsa_circ_0038249	chr16	18839352	18845760	exonic	SMG1

hsa_circRNA_100430	0.037870295	1.7259021	hsa_circ_0007167	chr1	203676136	203677232	exonic	ATP2B4
hsa_circRNA_065793	0.018383046	2.0784718	hsa_circ_0065793	chr3	50131152	50131308	exonic	RBM5
hsa_circRNA_007482	0.002718542	1.6548106	hsa_circ_0007482	chr9	137582757	137593179	exonic	COL5A1

circRNA: deregulated circRNA with greater intensity values in atherotrombotic stroke patients compared with cardioembolic; p-value: p-value calculated from unpaired t-test; FC (abs): absolute ratio (no log scale) of normalized intensities between two conditions; Alias: circRNA ID in circBase (http://circbase.mdc-berlin.de); Annotations, include chrom, strand, txStart, txEnd, circRNA_type and GeneSymbol; circRNA_type: The circRNAs are classified into 5 types: "exonic", "intronic", "antisense", "sense overlapping" and "intergenic". "Exonic" represents circRNA arising from the exons of the linear transcript; "Intronic" represents the circRNA arising from an intron of the linear transcript; "antisense" represents circRNA whose gene locus overlap with the linear RNA, but transcribed from the opposite strand; "sense overlapping" represents circRNA transcribed from same gene locus as the linear transcript, but not classified into "exonic" and "intronic"; "intergenic" represents circRNA located outside known gene locus.

Additional table S5. Upregulated expression of top 25 circRNAs in atherotrombotic versus undetermined stroke patients. 1.5 Fold up regulated circRNAs with a 0.05 p-value cut-off in the condition pair Atherotrombotic versus Cardioembolic.

circRNA	p-value	FC (abs)	Alias (circBase)	chromosome position			circRNA_type	GeneSymbol
hsa_circRNA_103433	0.040158153	1.6741177	hsa_circ_0005332	chr3	114069120	114070725	exonic	ZBTB20
hsa_circRNA_100837	0.010467779	3.0265662	hsa_circ_0022537	chr11	62594626	62595103	exonic	STX5
hsa_circRNA_043650	0.029390184	1.9536319	hsa_circ_0043650	chr17	39845126	39847898	exonic	EIF1
hsa_circRNA_100662	0.049526102	2.3475986	hsa_circ_0006654	chr10	102683731	102685776	exonic	FAM178A
hsa_circRNA_042882	0.046137568	2.1428329	hsa_circ_0042882	chr17	29483000	29533389	exonic	NF1
hsa_circRNA_000242	0.036117569	1.8598664	hsa_circ_0000242	chr10	70864370	70864480	antisense	SRGN
hsa_circRNA_013058	0.023426999	2.0270888	hsa_circ_0013058	chr1	85331067	85331842	exonic	LPAR3
hsa_circRNA_005801	0.020011767	1.562035	hsa_circ_0005801	chr10	98303832	98325183	exonic	TM9SF3
hsa_circRNA_101643	0.010757841	1.5392954	hsa_circ_0036750	chr15	90446447	90454051	exonic	ARPIN
hsa_circRNA_090183	0.0208035	1.7578911	hsa_circ_0090183	chrX	37265461	37285253	exonic	PRRG1
hsa_circRNA_067574	0.028412134	1.9753255	hsa_circ_0067574	chr3	140978964	141006295	exonic	PXYLP1
hsa_circRNA_002781	0.011514579	2.897959	hsa_circ_0002781	chr9	26861063	26884883	exonic	CAAP1
hsa_circRNA_401782	0.012010082	1.5371521		chr17	34144719	34149837	exonic	TAF15
hsa_circRNA_406412	0.006671561	1.6307879		chr3	186517507	186517774	intronic	RFC4
hsa_circRNA_407205	0.0226581	3.5466576		chr9	99481790	99483412	sense overlapping	RP11-535M15.1
hsa_circRNA_000950	0.009298183	1.8616349	hsa_circ_0001525	chr5	131761180	131761386	intronic	C5orf56
hsa_circRNA_402150	0.001776501	2.725805		chr2	1093881	1168869	exonic	SNTG2
hsa_circRNA_401844	0.024848115	2.4560364		chr17	57963443	57968289	exonic	TUBD1
hsa_circRNA_000855	0.035275421	1.5458879	hsa_circ_0000632	chr15	74927307	74929309	sense overlapping	EDC3
hsa_circRNA_030997	0.017515384	1.6520184	hsa_circ_0030997	chr13	113960799	113977741	exonic	LAMP1
hsa_circRNA_000646	0.036540206	1.6835728	hsa_circ_0000646	chr15	83819966	83832827	exonic	HDGFRP3
hsa_circRNA_405627	0.038150374	1.7281144		chr17	73170846	73177283	exonic	SUMO2

hsa_circRNA_404480	0.00368756	1.5353523		chr1	38300748	38305830	sense overlapping	MTF1
hsa_circRNA_071668	0.001287408	1.5677239	hsa_circ_0071668	chr5	843714	843839	exonic	ZDHHC11
hsa_circRNA_080099	0.03981788	2.5891074	hsa_circ_0080099	chr7	45011271	45016256	exonic	MYO1G

circRNA: deregulated circRNA with greater intensity values in atherotrombotic stroke patients compared with undetermined; p-value: p-value calculated from unpaired t-test; FC (abs): absolute ratio (no log scale) of normalized intensities between two conditions; Alias: circRNA ID in circBase (http://circbase.mdc-berlin.de); Annotations, include chrom, txStart, txEnd, circRNA_type and GeneSymbol; circRNA_type: The circRNAs are classified into 5 types: "exonic", "intronic", "antisense", "sense overlapping" and "intergenic". "Exonic" represents circRNA arising from the exons of the linear transcript; "Intronic" represents the circRNA arising from an intron of the linear transcript; "antisense" represents circRNA whose gene locus overlap with the linear RNA, but transcribed from the opposite strand; "sense overlapping" represents circRNA transcribed from same gene locus as the linear transcript, but not classified into "exonic" and "intronic"; "intergenic" represents circRNA located outside known gene locus.

Additional table S6. Downregulated expression of top 25 circRNAs in atherotrombotic versus undetermined stroke patients. 1.5 Fold up regulated circRNAs with a 0.05 p-value cut-off in the condition pair Atherotrombotic versus Undetermined.

circRNA	p-value	FC (abs)	circRNA	Alias (circBase)	chromosome position			circRNA_type	GeneSymbol
hsa_circRNA_100018	0.003066785	2.4415166	hsa_circRNA_100018	hsa_circ_0009361	chr1	1749275	1770677	exonic	GNB1
hsa_circRNA_403834	0.002028034	1.7909661	hsa_circRNA_403834		chr7	72863871	72865324	exonic	BAZ1B
hsa_circRNA_092416	0.047104691	1.5574844	hsa_circRNA_092416	hsa_circ_0000593	chr15	41988272	42005694	exonic	MGA
hsa_circRNA_006314	0.007978786	1.8727172	hsa_circRNA_006314	hsa_circ_0006314	chr15	72810407	72837305	exonic	ARIH1
hsa_circRNA_406313	0.002471054	1.7647655	hsa_circRNA_406313		chr3	100348441	100438902	sense overlapping	TFG
hsa_circRNA_004646	0.01479424	1.6908195	hsa_circRNA_004646	hsa_circ_0004646	chr1	162546566	162567648	exonic	UAP1
hsa_circRNA_075625	0.016361863	1.8534081	hsa_circRNA_075625	hsa_circ_0075625	chr6	10935290	10956475	exonic	SYCP2L
hsa_circRNA_001543	0.041581839	1.5858897	hsa_circRNA_001543	hsa_circ_0001720	chr7	77378740	77387395	sense overlapping	RSBN1L
hsa_circRNA_068697	0.010798495	1.575369	hsa_circRNA_068697	hsa_circ_0068697	chr3	196846280	196869639	exonic	DLG1
hsa_circRNA_100085	0.017153672	1.906965	hsa_circRNA_100085	hsa_circ_0005075	chr1	21377358	21415706	exonic	EIF4G3
hsa_circRNA_100086	0.003670822	2.048826	hsa_circRNA_100086	hsa_circ_0000026	chr1	21377358	21437876	exonic	EIF4G3
hsa_circRNA_007335	0.016302117	1.6057207	hsa_circRNA_007335	hsa_circ_0007335	chr10	76978820	76979114	exonic	VDAC2
hsa_circRNA_104425	0.002791101	1.6939598	hsa_circRNA_104425	hsa_circ_0081006	chr7	91842508	91855996	exonic	KRIT1
hsa_circRNA_100604	0.047075566	1.5533658	hsa_circRNA_100604	hsa_circ_0009172	chr10	70218860	70229920	exonic	DNA2
hsa_circRNA_028152	0.02016626	2.0837959	hsa_circRNA_028152	hsa_circ_0028152	chr12	110397651	110399490	exonic	GIT2
hsa_circRNA_083776	0.039776445	1.5375104	hsa_circRNA_083776	hsa_circ_0083776	chr8	27514298	27530537	exonic	SCARA3
hsa_circRNA_083946	0.026422628	1.6387149	hsa_circRNA_083946	hsa_circ_0083946	chr8	37732032	37735069	exonic	RAB11FIP1
hsa_circRNA_104126	0.037379623	1.8526072	hsa_circRNA_104126	hsa_circ_0076798	chr6	53365044	53365148	exonic	GCLC
hsa_circRNA_100848	0.012341721	1.7579572	hsa_circRNA_100848	hsa_circ_0022812	chr11	65055186	65063461	exonic	POLA2
hsa_circRNA_006023	0.012460862	1.560156	hsa_circRNA_006023	hsa_circ_0006023	chr2	27900161	27900791	exonic	SLC4A1AP
hsa_circRNA_103639	0.016559004	1.8737968	hsa_circRNA_103639	hsa_circ_0007646	chr4	52729602	52758017	exonic	DCUN1D4
hsa_circRNA_081379	0.009090016	1.7494497	hsa_circRNA_081379	hsa_circ_0081379	chr7	99621041	99621555	exonic	ZKSCAN1

hsa_circRNA_401253	0.01311697	1.7559226	hsa_circRNA_401253		chr14	55457930	55467710	exonic	WDHD1
hsa_circRNA_405870	0.013868434	1.6725316	hsa_circRNA_405870		chr2	44683706	44776867	intronic	CAMKMT
hsa_circRNA_003898	0.001182297	1.6992846	hsa_circRNA_003898	hsa_circ_0003898	chr2	231624673	231658046	exonic	CAB39

circRNA: deregulated circRNA with greater intensity values in atherotrombotic stroke patients compared with undetermined p-value: p-value calculated from unpaired t-test; FC (abs): absolute ratio (no log scale) of normalized intensities between two conditions; Alias: circRNA ID in circBase (http://circbase.mdc-berlin.de); Annotations, include chrom, txStart, txEnd, circRNA_type and GeneSymbol; circRNA_type: The circRNAs are classified into 5 types: "exonic", "intronic", "antisense", "sense overlapping" and "intergenic". "Exonic" represents circRNA arising from the exons of the linear transcript; "Intronic" represents the circRNA arising from an intron of the linear transcript; "antisense" represents circRNA whose gene locus overlap with the linear RNA, but transcribed from the opposite strand; "sense overlapping" represents circRNA transcribed from same gene locus as the linear transcript, but not classified into "exonic" and "intronic"; "intergenic" represents circRNA located outside known gene locus.

Additional table S7. Up and downregulated expression of circRNAs in cardioembolic versus undetermined stroke patients. 1.5 Fold up regulated circRNAs with a 0.05 p-value cut-off in the condition pair Cardioembolic versus Undetermined.

circRNA	p-value	FC (abs)	Regulation	Alias (circBase)	chromosome positions			circRNA_type	GeneSymbol
hsa_circRNA_405627	0.042319481	1.5146971	up		chr17	73170846	73177283	exonic	SUMO2
hsa_circRNA_400020	0.026813857	1.8052207	up	hsa_circ_0092372	chr11	9450336	9450536	intronic	IPO7
hsa_circRNA_101940	0.028829636	1.832913	up	hsa_circ_0004018	chr17	1703150	1704318	exonic	SMYD4
hsa_circRNA_402533	0.044157744	1.8186796	up		chr20	18022177	18022367	exonic	OVOL2
hsa_circRNA_406174	0.008380548	1.953653	up		chr22	28249524	28269803	sense overlapping	PITPNB
hsa_circRNA_402283	0.04050379	1.7096854	up		chr2	62227835	62228117	exonic	COMMD1
hsa_circRNA_008267	0.023016747	1.8975145	up	hsa_circ_0008267	chr3	195415403	195416309	exonic	LINC00969
hsa_circRNA_051778	0.043541928	1.50812	up	hsa_circ_0051778	chr19	49298318	49303095	exonic	BCAT2
hsa_circRNA_101655	0.00786997	1.5889352	down	hsa_circ_0000660	chr15	94899365	94945248	exonic	MCTP2
hsa_circRNA_055953	0.002207237	1.6670279	down	hsa_circ_0055953	chr2	107446521	107450602	exonic	ST6GAL2
hsa_circRNA_003904	0.018948933	1.6320684	down	hsa_circ_0003904	chr1	29585016	29587415	exonic	PTPRU
hsa_circRNA_101563	0.034684222	1.5473558	down	hsa_circ_0035944	chr15	66021409	66031213	exonic	DENND4A
hsa_circRNA_104781	0.011427264	1.6665397	down	hsa_circ_0087063	chr9	37425918	37426651	exonic	GRHPR
hsa_circRNA_000620	0.000694352	2.4963159	down	hsa_circ_0000726	chr16	89484691	89492069	sense overlapping	ANKRD11
hsa_circRNA_403068	0.017950543	1.5190044	down		chr4	289226	289944	exonic	ZNF732
hsa_circRNA_005131	0.000319183	1.5503095	down	hsa_circ_0005131	chr1	156294762	156304709	exonic	CCT3
hsa_circRNA_004566	0.000946486	2.43209	down	hsa_circ_0004566	chr16	89484691	89488713	sense overlapping	ANKRD11

circRNA: deregulated circRNA with greater/lower intensity values in cardioembolic stroke patients compared with undetermined; p-value: p-value calculated from unpaired t-test; FC (abs): absolute ratio (no log scale) of normalized intensities between two conditions; Alias: circRNA

ID in circBase (http://circbase.mdc-berlin.de); Annotations, include chrom, txStart, txEnd, circRNA_type and GeneSymbol; circRNA_type: The circRNAs are classified into 5 types: "exonic", "intronic", "antisense", "sense overlapping" and "intergenic". "Exonic" represents circRNA arising from the exons of the linear transcript; "Intronic" represents the circRNA arising from an intron of the linear transcript; "antisense" represents circRNA whose gene locus overlap with the linear RNA, but transcribed from the opposite strand; "sense overlapping" represents circRNA transcribed from same gene locus as the linear transcript, but not classified into "exonic" and "intronic"; "intergenic" represents circRNA located outside known gene locus.
